# *Serratia nevei* in Nigeria: First Report and Global Distribution

**DOI:** 10.3390/microorganisms13122732

**Published:** 2025-11-29

**Authors:** Ayodele Timilehin Adesoji, Emmanuel Dayo Alabi, Vittoria Mattioni Marchetti, Roberta Migliavacca

**Affiliations:** 1 Department of Microbiology, Federal University, Dutsin-Ma 821101, Katsina State, Nigeria; timmyayus2002@yahoo.com (A.T.A.); demmanuel@fudutsinma.edu.ng (E.D.A.); 2 Microbiology and Clinical Microbiology Unit, Scienze Clinico, Chiurgiche, Diagnostiche e Pediatriche Department, University of Pavia, 27100 Pavia, Italy; roberta.migliavacca@unipv.it; 3 Istituto di Ricovero e Cura a Carattere Scientifico Policlinico S. Matteo, 27100 Pavia, Italy

**Keywords:** *Serratia nevei*, epidemiology, whole-genome sequencing

## Abstract

*Serratia* species are opportunistic human pathogens found in diverse environmental habitats. Here, we report the first isolation of *Serratia nevei* from food samples in Nigeria. During a two-month epidemiological surveillance at a local food market in Dutsin-Ma, Katsina State, Nigeria, a total of 180 food samples were collected, and isolation and species identification were performed using chromogenic agar and MicroScan autoSCAN-4, respectively. Antimicrobial susceptibility and minimum inhibitory concentrations (MICs) were determined using the MicroScan autoSCAN-4 system. Strain F129B, recovered from a fresh, unprocessed beef sample, was initially identified as *Klebsiella pneumoniae* by chromogenic agar and MicroScan autoSCAN-4, and subsequently as *Serratia marcescens* by MALDI-TOF MS. Only Whole Genome Sequencing (WGS) and bioinformatics analyses confirmed its identity as *S. nevei*. The strain was then selected for further characterization by Whole Genome Sequencing (WGS) and bioinformatics analyses to confirm its identity. The strain was phenotypically resistant to amoxicillin/clavulanic acid and colistin, with elevated MICs for aztreonam (4 mg/L) and cefuroxime (16 mg/L). In silico analyses of its genome confirmed the isolate as *S. nevei*, harboring genes conferring resistance to β-lactams (*blaSTR-2*), aminoglycosides (*aac* (*6′*)*-Ic*), fosfomycin (*fosA*), streptomycin (*satA*), and tetracycline (*tet* (*41*)). Its virulence repertoire comprises genes associated with adhesion (*yidE*, *yidR*, *yidQ*), colicin tolerance (*creA* and *creD*), and heavy metal resistance (*czcD*, *chrBACF* operon). These findings underscore the need for genomic characterization for accurate species identification within the *Serratia* genus. Our findings revealed the emergence of *S. nevei* in the food supply chain and highlighted its potential for zoonotic transmission. Robust surveillance of the local food supply chain is urgently needed in north-western Nigeria.

## 1. Introduction

The genus *Serratia* consists of ubiquitous Gram-negative bacteria and belongs to the *Yersiniaceae* family [1]. The genus *Serratia* has been found in diverse environmental niches and comprises more than ten different species [2,3,4,5]. Recently, *Serratia nevei* was described by Cho and colleagues and included in the taxonomy of *Serratia* species [6]. In fact, Cho and colleagues recovered *S. nevei* from fresh farm produce (cucumber and basil) in Germany’s retail market, veterinary samples in Thailand, and clinical samples in Spain [6,7,8]. The impact of *Serratia* species on antimicrobial resistance has grown substantially over time, with a notable surge in 2020. This impact has been facilitated by plasmid-mediated transmission of genes encoding extended-spectrum beta-lactamases (ESBLs) and carbapenemases from other Enterobacterales [9]. Indeed, *S. nevei* has been reported to acquire clinically relevant antibiotic resistance traits, including genes conferring resistance to carbapenems. Additionally, colistin-resistant strains have also been documented [8,9]. However, frequent misidentification of species within the *Serratia* genus complicates surveillance efforts, contributing to persistent gaps in knowledge regarding its spread [10]. Nigeria has recently undertaken epidemiological surveillance studies on antibiotic-resistant Enterobacterales, focusing to a lesser extent on secondary Enterobacterales such as *Serratia* spp. [11].

Here, we report the first isolation of *S. nevei* in Nigeria and determine its antibiotic-resistant profiles, resistome, virulome, and its genomic relatedness to other *S. nevei* strains, thereby, providing insights into the global dissemination patterns of this uncommon species.

## 2. Materials and Methods

### 2.1. Strain Isolation, Preliminary Identification, and Antibiotic Suceptibility Tests

An epidemiological study was conducted in Dutsin-Ma town, Katsina State, Nigeria, between October and November 2022, to investigate the circulation of *Klebsiella* spp. strains within the area. A total of 300 samples were collected from three sampling sites: the Wednesday Market (the main market of the city), Malam Mande General Hospital, and the Comprehensive Health Care Centre. Of the total samples, 180 were obtained from food and animal sources, while 120 were collected from human clinical specimens. The food samples comprised locally processed ready-to-eat products, including balangu meat (*n* = 15) and suya meat (*n* = 15), as well as tubers (cassava, *n* = 10), fruits (garden egg, *n* = 10), and vegetables (cabbage, *n* = 20; spinach, *n* = 20; cucumber, *n* = 10). Animal samples consisted of raw beef meat (*n* = 55) and goat meat (*n* = 25) [12].

Bacterial isolation was performed on the collected samples using MacConkey and CHROM agar (Scharlab Italia s.r.l., Lodi, Italy). The bacterial isolates were subsequently shipped to the Department of Clinical-Surgical, Diagnostic, and Pediatric Sciences, Unit of Microbiology and Clinical Microbiology, at the University of Pavia, Italy, for further analysis.

At Pavia, species identification and analysis of the antimicrobial susceptibility patterns of the bacterial isolates were performed using the MicroScan autoSCAN-4 (Beckman Coulter, Brea, CA, USA) semi-automated system. The identified species were further confirmed using MALDI-TOF MS (Bruker Daltonics GmbH, Billerica, MA, USA) and analyzed using BioTyper version 3.0. The minimum inhibitory concentrations (MICs) obtained were interpreted following EUCAST breakpoints (2024).

### 2.2. Whole Genome Sequencing and Bioinformatics Analysis

One of the isolates (Strain ID: F129B), recovered on 27 October 2022, from beef, which was initially identified as *Klebsiella pneumonia* with MicroScan autoSCAN-4 but later identified as *Serratia marcescens* by MALDI-TOF-MS, was further analyzed for identity confirmation with Whole Genome Sequencing (WGS) and bioinformatic analysis due to this diagnostic discrepancy. The genomic DNA of F129B was extracted using the DNeasy Blood & Tissue kit (Qiagen, Hilden, Germany) and sequenced on an Illumina HiSeq PE150 platform (Illumina Inc., San Diego, CA, USA) with 350 bp paired-end sequencing, following library preparation with the Nextera XT library preparation kit. Reads were assembled within the Shovill pipeline, discharging short contigs with low coverage or pure homopolymers (https://github.com/tseemann/shovill, accessed on 12 September 2025). Assembled sequences were annotated using Rapid Annotation using Subsystems Technology (RAST server) [13]. The resistome and plasmid replicon content were determined by uploading the assembled sequences to ResFinder 4.1 and PlasmidFinder v2.1, respectively, available on the Center for Genomic Epidemiology website (https://www.genomicepidemiology.org/, accessed on 12 September 2025). Virulence determinants were evaluated using the SEED viewer, available on the RAST server [13]. To understand the virulome evolution and the potential of F129B in causing infections, evaluation of phage sequences was obtained using the online tool PHASTER (PHAge Search Tool Enhanced Release) (https://phaster.ca, accessed on 12 September 2025), while the phage classification was assessed through the online tool PhaBOX 2.0v [14,15,16]. Part-of-whole graphs and timeline depictions were represented using GraphPad Prism version 10.0.0 for Macintosh, GraphPad Software, Boston, MA, USA (www.graphpad.com, accessed on 12 September 2025). A Sankey diagram was created using SankeyMATIC (https://sankeymatic.com/about/, accessed on 12 September 2025).

### 2.3. Genomic Species Identification

Average nucleotide identity (ANI) analysis was performed using the FastANI tool (https://github.com/ParBLiSS/FastANI, accessed on 12 September 2025), whereas the digital DNA-DNA hybridization (dDDH) values were calculated using formula 2 in the Genome-to-Genome Distance Calculator version 2.1 [4,17,18].

### 2.4. Phylogenetic Analysis

Phylogenetic relationships were determined for the studied strain using the available 163 genomes of *S. nevei* retrieved from the NCBI database. SNP-based phylogeny was depicted using Parsnp v 2.0.5 (https://github.com/marbl/parsnp, accessed on 12 September 2025) using *S. nevei* LMG 31536 (GCA_037948395.1) as the reference genome [19]. Metadata on the 163 genomes are available in Supplementary Table S1. Clonality cut-off was set at SNPs ≤ 25 [20]. A graphic illustration of the trees was built with the Interactive Tree Of Life (iTOL) (https://itol.embl.de/, accessed on 12 September 2025). Clustering of the genetic sequences was performed on the total pool of *S. nevei* using the FastBaps algorithm [21]. The resistome of the 163 genomes was identified using ResFinder 4.1.

### 2.5. Data Availability

The nucleotide sequence of F129B was deposited and is available in GenBank under the Bioproject PRJNA1135675 (Biosample: SAMN42488087).

## 3. Results

Among the 180 food samples collected, 30 (16.7%) were recovered as presumptive *Klebsiella pneumoniae* isolates through preliminary species characterization using the MicroScan Autoscan system [10]. In detail, 20/30 were classified as *K. pneumoniae*, 5/30 *Klebsiella quasipneumoniae* subsp. *similipneumoniae*, 3/30 *Klebsiella variicola* subsp. *variicola*, 1/30 *Klebsiella michiganensis,* and the remaining 1/30 were *K. pneumoniae* but at lower ID values (ID < 85%). The isolate F129B underwent further investigation.

Unexpectedly, FI29B was later identified to belong to the *S. marcescens* species by MALDI-TOF MS with a low confidence score (<2.0). The prevalence of *Serratia* spp. was 0.6% (1/180) among all the samples collected. The antibiotic susceptibility profiles of the selected isolate (F129B) revealed resistance (R) to amoxicillin/clavulanic acid (>32 mg/L) and colistin (>4 mg/L R). Colistin resistance was expected as an intrinsic trait of *Serratia* spp. Additionally, increased MIC values were observed for aztreonam (4 mg/L) and cefuroxime (16 mg/L).

### 3.1. WGS and In Silico Analysis

**Figure 1:**
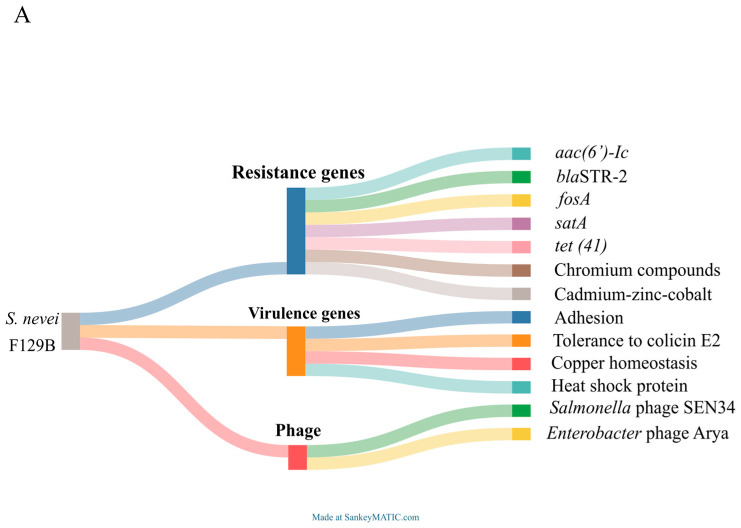

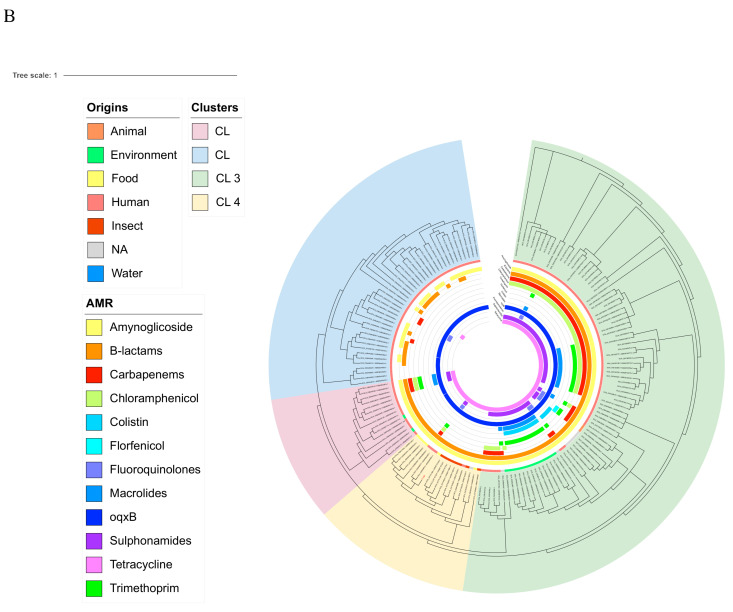
(**A**) Sankey diagram picturing resistome, virulome, and phage content of F129B. Diagram created using SankeyMATIC (https://sankeymatic.com/about/, accessed on 12 September 2025). *Aac*(*6′*)*-Ic* = aminoglycoside resistance gene, *bla*_STR-2_ = β-lactam resistance gene, *fosA* = fosfomycin resistance gene, *satA* = streptomycin, *tet*(*41*) = tetracycline resistance gene. (**B**) iTOL v6 representation of SNPs-based phylogeny for the 164 available genomes, obtained by parsnp v 2.0.5, and the related origin and resistome. F129B is colored in red. AMR = antimicrobial resistance genes (grouped and colored by classes of antibiotics). The AMR refers to the presence of at least one resistance gene of the corresponding antibiotic class in the resistome.

The assembled genome of strain F129B comprises 5,369,846 bp with a genome coverage of 120×, and contains 5019 protein-coding genes, with a G+C content of 59.7%. Interestingly, the bioinformatic evaluation pointed out a different species. In fact, the highest FastANI and dDDH^4^ values for F129B were established with the reference genomes *S. nevei* LMG 31536 (GCA_037948395.1) (98.77% and 90.6%, respectively), instead of *S. marcescens*. F129B possessed a narrow resistome composed of aminoglycoside (*aac* (*6′*)*-Ic*), β-lactam (*bla*_STR-2_), fosfomycin (*fosA*), streptomycin (*satA*), and tetracycline (*tet* (*41*)) resistance genes, confirming the antimicrobial susceptibility profile obtained (Figure 1A). Moreover, F129B harbored resistance traits to chromium compounds (*chrBACF* operon) and cadmium–zinc–cobalt (*czcD*). No acquired genes for the colistin resistance phenotype were pointed out. The resistome identified did not explain the susceptibility profile obtained. Its virulome included genes associated with adhesion (*yidE*, *yidR*, *yidQ*, which are involved in hyper-adhesion and biofilm formation), tolerance to colicin E2 (*creA* and *creD*, producing periplasmic and inner membrane protein), copper homeostasis (*scsABCD*, *cutADEF*, *copC*, and *copD*), and the 16 kDa Heat shock protein A/B (Figure 1A). No plasmid elements were detected. Interestingly, four phage regions were detected within the F129B chromosome: a 43.8 kb region carrying the virulence genes *copC* and *copD* and showing similarities with the *Salmonella* phage SEN34 (GenBank accession number: NC_028699.1), a 41.1 kb region with identities with the phage *Enterobacter* phage Arya (GenBank Accession number: NC_031048.1), a 9.3 kb and a 9.5 kb regions similar to *Salmonella* SPN3UB (NC_019545.1), and *Klebsiella* phage ST13-OXA48phi12.1 (GenBank Accession number: NC_ 049453.1). The phage classification indicated a temperate phage for all regions, except for the 9.5 kb region which was assigned as virulent (Figure 1A).

### 3.2. Genomic Relatedness

The SNP-based phylogenetic tree and the FastBaps algorithm revealed that *S. nevei* is globally structured in four distinct clusters (CL1-4) (Figure 1B). F129B fell within CL4 and clustered with GCA_039409615.1 and GCA_038444875.1, both collected in 2022 from human samples in Thailand and Spain, respectively. This clustering was confirmed by SNP count, with the lowest values observed for GCA_039409615.1 (SNPs *n* = 9301) and GCA_038444875.1 (SNPs *n* = 9513). F129B itself revealed identity with other *S. nevei* genomes, supported by the high number of SNPs (>9000). However, it exhibited the highest relatedness with GCA_029960205.1 (SNPs *n* = 9609), collected in 2022 from a human sample in Ecuador, as well as with GCA_008364245.1 (SNPs *n* = 9832), GCA_037948395.1 (SNPs *n* = 9832), GCA_008011825.1 (SNPs *n* = 9833), and GCA_008364335.1 (SNPs *n* = 9833), all isolated in 2015 from food samples in Germany (Figure 1B). These similarities suggest widespread dissemination of the infrequent *S. nevei*, highlighting its ability to adapt to both animal and human niches.

Evaluation of the *S. nevei* resistome revealed diverse compositions of antimicrobial resistance genes across the four clusters. Specifically, the *S. nevei* population was primarily associated with resistance to aminoglycosides, β-lactams, and tetracyclines, while CL3 showed a high propensity for acquiring carbapenem and sulphonamide resistance genes (Figure 1B), a trait scarce or absent in the remaining clusters.

**Figure 2:**
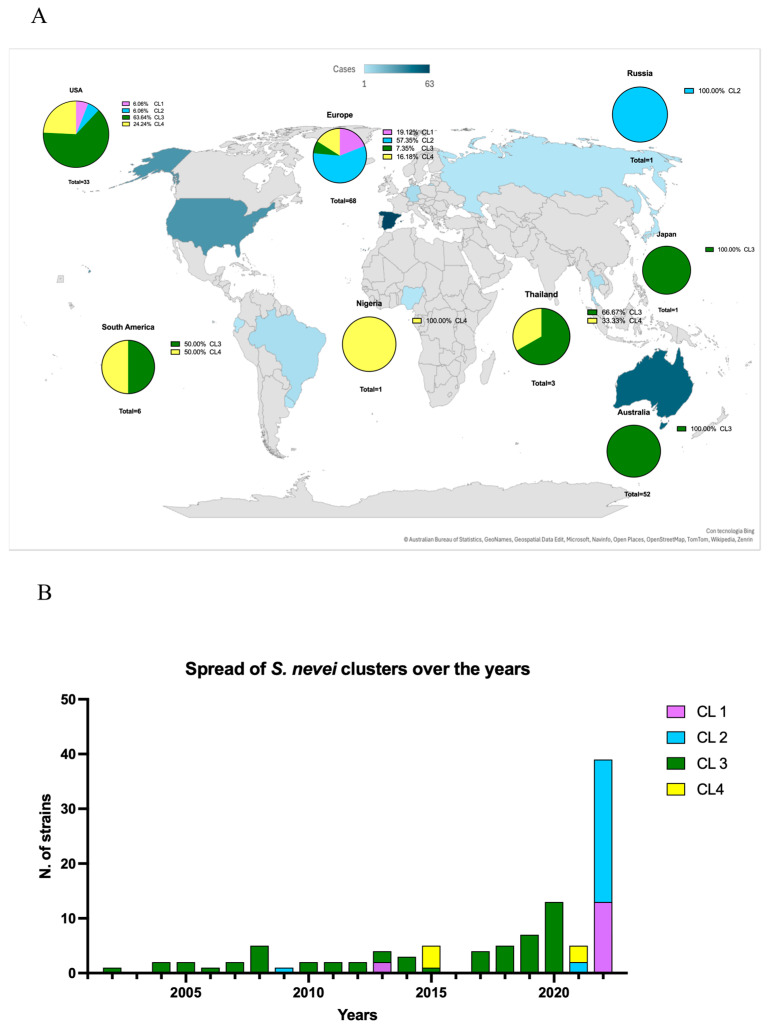
(**A**) epidemiological map of *S. nevei* spread. In the pie chart: purple = cluster 1 (CL1); light blue = cluster 2 (CL2); green = cluster 3 (CL3); yellow = cluster 4 (CL4). The countries are colored based on the number of *S. nevei* genomes retrieved from NCBI. Pie charts elucidate the precise number of genomes related and the number of different clusters is expressed in percentage. (**B**) distribution of *S. nevei* genomes based on the year of isolation (*n* = 144/164). Purple = cluster 1 (CL1); light blue = cluster 2 (CL2); green = cluster 3 (CL3); yellow = cluster 4 (CL4). In the present graph, strains with no available metadata on the year of isolation have not been included (*n* = 13/20). Genomes with unknown metadata are not included in the depiction (*n* = 7/20). Timeline created by GraphPad v10.3.1.

Across the entire *S. nevei* population, the four clusters exhibited differences in their genomic size and geographic distribution. Numerically, CL3 (*n* = 84) and CL2 (*n* = 42) are the dominant clusters, followed by CL4 (*n* = 23), and then CL1 (*n* = 15). Geographically, CL3 showed a heterogeneous distribution, occurring in Australia, the USA, Japan, Spain, and Thailand. Similarly, CL4 was detected in Nigeria (in this study), as well as in Germany, Uruguay, Brazil, Ecuador, the USA, Spain, and Thailand. In contrast, CL1 and CL2 exhibit a more homogeneous distribution, primarily restricted to Spain, with sporadic occurrence in the USA (Figure 2A).

Temporally, CL3 emerged in 2002 and persisted steadily until 2022, while CL4 appeared sporadically in 2006 before increasing in prevalence in 2015, 2021, and 2022 (Figure 2B).

## 4. Discussion

*S. nevei* is an uncommon pathogen that has been reported in specific geographic regions, including Spain and Germany, in both clinical and environmental settings [6,7]. Here, we report the first recovery of *S. nevei* from beef in Nigeria. Strain F129B exhibited low antimicrobial resistance traits, but possessed significant virulence factors, particularly related to adhesion, resistance to heavy metals, and copper homeostasis. Additionally, the 16 kDa heat shock proteins A and B harbored by the strain play a role in stress response, virulence, and defense in *Serratia* sp. TEL [22]. The presence of virulence gene-mediating pathways involving adhesion and copper homeostasis highlights the capacity and possibility of *S. nevei* emerging as a moderately virulent microorganism. Studies on the actual virulence of *S. nevei* are absent from the literature and require further investigation.

The apparent absence of mobile elements, such as plasmids, suggest that F129B has a limited propensity to acquire additional external traits, such as resistance and virulence genes. However, phage regions were identified within the F129B chromosome. Phages, also known as bacteriophages, are viruses that can infect bacteria. Phages that are classified as temperate can integrate within the bacterium’s host chromosome, whereas virulent phages can immediately induce bacterium cell destruction through lysis [23]. Temperate phages may provide a mutual evolutionary advantage to the bacteria by allowing the introduction of additional genes, such as virulence and antibiotic resistance genes [24]. The presence of phage elements in *S. nevei* suggests a certain plasticity of its genome, with the possibility of acquiring new, evolutionarily favorable traits.

The absence of genes encoding ESBL does not diminish the clinical relevance of *S. nevei*, as the isolate carries the chromosomal β-lactamase-encoding gene *bla_SRT-2_*. In *S. marcescens*, *bla_SRT-2_* is often found alongside other β-lactam resistance genes, including *bla_CTX-M-3_*, *bla_TEM-1_*, *bla_KPC-2_*, and the aminoglycoside resistance gene *aac*(*6′*)*-Ic* [25,26,27]. The *S. nevei* strain we characterized in this study harbored only *aac*(*6′*)*-Ic* with *bla_SRT-2_*, but cases of carbapenemase-producing *S. nevei* have been already reported in the scientific literature. During wastewater treatment plant surveillance in Georgia, USA, Hassan and coauthors identified seven high-level colistin-resistant *S. nevei* strains carrying *mcr-9* resistance genes [27]. Moreover, pan-drug-resistant *S. nevei* has recently been reported in veterinary settings. Leelapsawas and co-authors were the first to describe the occurrence of the clinically relevant carbapenemase OXA-181 in two *S. nevei* strains of canine origin, highlighting the species’ ability to acquire relevant antimicrobial-resistant traits [7]. Furthermore, an emerging *S. nevei* and VIM producer has been highlighted in a large Intensive Care Unit (ICU) in Spain. Over the last 20 years, it has been identified as a source of ESBL outbreaks and carbapenemase-producing *Enterobacterales* [8]. The precise effect of *S. nevei* on humans is unclear and needs further investigation. However, its ability to adapt to human, veterinary, and aquatic environments may pose a challenge to global public health due to its propensity to acquire antimicrobial resistance and virulence factors.

The phylogenetic and clustering analysis highlighted a heterogeneous feature within the *S. nevei* population. F129B fell in cluster CL4, which showed a global circulation, but to a lesser extent than when compared to CL3, which seemed to be the most representative cluster. Moreover, F129B had a narrow resistome, excluding carbapenemase genes, which were common within CL4. These findings demonstrate the genomic differences within the *S. nevei* population, potentially allowing for the development of MDR or PDR phenotypes and their implication in infectious diseases in both animals and humans. However, these speculations require confirmation through further analyses and specific virulence assessment experiments.

Definitive species identification undoubtedly makes the overall characterization of *S. nevei* difficult. To date (21 November 2025), in the scientific literature there are a total of 14 papers available reporting on *S. nevei* strains in different niches (Table 1). As reported in the literature, precise identification and species differentiation is only possible through molecular approaches along with the use of bioinformatics tools. As in this study, precise identification is achieved using omics techniques such as WGS, while routine systems based on culture growth or mass spectrometry have proved to be inadequate. The frequent misidentification of *S. nevei* as *S. marcescens* further complicates efforts to track its spread, resulting in persistent gaps in knowledge about its dissemination.

## 5. Conclusions

In conclusion, this report confirms the first isolation of *S. nevei* from food in Nigeria and highlights the importance of WGS, not only for definitive identification but also for revealing important virulence genes and phage elements. These findings further highlight the importance of improved species identification and molecular characterization within the *Serratia* genus, due to its epidemiological and clinically relevant features. In the context of the One-Health approach, bacterial species previously considered minor require surveillance to prevent their evolution into reservoirs of emerging (new) infectious diseases, as environmental, animal, and human interactions can facilitate pathogen spillover and adaptation. Given that only a single isolate of *S. nevei* was incidentally recovered during the surveillance of *Klebsiella* species in the local food supply chain, the findings do not provide a comprehensive representation of this species’ distribution in the region. This limitation is acknowledged in the study. In order to better understand the real distribution of *S. nevei*, this microorganism should be included in passive surveillance programs within different ecological niches.

## Figures and Tables

**Table 1:** Information on the *S. nevei* publications available on 21 November 2025.

Author	Source of Isolation	Country	Identification Method	DOI
Cho et al., 2020 [6]	Cucumber	Germany	16S rRNA, DNA-DNA hybridization	https://doi.org/10.1016/j.syapm.2020.126055
Abreo et al., 2021 [28]	Locusts	Uruguay	gyrB and ANI	https://doi.org/10.1016/j.syapm.2020.126177
Pérez-Viso et al., 2024 [9]	Bloodstream infections	Spain	Taxonomic Sequence Classification System Kraken and PATO package in R, calculating the MASH distance	https://doi.org/10.1128/spectrum.02762-23
Leelapsawas et al., 2024 [7]	Canine infection	Thailand	ANI	https://doi.org/10.1128/spectrum.03589-23
Aracil-Gisbert et al., 2024 [8]	ICU sink and clinical samples	Spain	16S rRNA and DNA-DNA hybridization	https://doi.org/10.1128/mbio.03054-23
Yaikhan et al., 2024 [29]	Clinical samples	Thailand	ANI	https://doi.org/10.3390/antibiotics13060531
Hassan et al., 2025 [27]	Raw sewage and water	USA	WGS	https://doi.org/10.1016/j.envpol.2024.125515
Ota et al., 2024 [30]	Hospital wastewater	Japan	GTDB Toolkit Classify v2.1.0	https://doi.org/10.3390/antibiotics13121122
Kang et al., 2025 [31]	Food	Canada	VITEK MS system (bioMérieux) and WGS	https://doi.org/10.1128/mra.00301-25
Liu et al., 2025 [32]	Clinical samples	USA, Canada, Romania, China, South Africa, Bulgaria	MALDI (as *S. marcescens*) and TYGS platform	https://doi.org/10.1016/j.crmicr.2025.100436
Zhu et al., 2025 [33]	Clinical samples from tertiary teaching hospital	China	dDDH and FastANI v1.34	https://doi.org/10.1016/j.crmicr.2025.100456
Peirano et al., 2025 [34]	Clinical samples	South Africa, Romania	TYGS and ANI	https://doi.org/10.1007/s10096-025-05254-x
Furlan et al., 2025 [35]	Wastewater	Brazil	ANI and dDDH	https://doi.org/10.1128/spectrum.01378-25
Zhu et al., 2025 [1]	Intensive Unit Care patients	China	Genome Database Taxonomy (GTDB)	https://doi.org/10.3389/fcimb.2025.1672468

## Data Availability

The data presented in this study are openly available in the GenBank repository at https://www.ncbi.nlm.nih.gov/bioproject/PRJNA1135675 (accessed on 25 September 2025), reference number SAMN42488087.

## Supplementary Materials

The following supporting information can be downloaded at: https://www.mdpi.com/article/10.3390/microorganisms13122732/s1, Table S1: Metadata on the 163 *S. nevei* genomes retrieved from NCBI database.
